# The complete mitochondrial genome of *Psyttalia incisi* (Silvestri, 1916) (Hymenoptera: Braconidae)

**DOI:** 10.1080/23802359.2022.2081942

**Published:** 2022-06-14

**Authors:** Deqing Yang, Xuxing Hao, Lili Jiang, Tsunglin Chou, Xiang Lin, Guoqing Yue, Kang Xiao, Jia Lin, Qinge Ji, Pumo Cai

**Affiliations:** a Institute of Biological Control, Plant Protection College, Fujian Agriculture and Forestry University, Fuzhou, China; b Key Laboratory of Biopesticide and Chemical Biology, Ministry of Education, Fuzhou, China; c State Key Laboratory of Ecological Pest Control for Fujian and Taiwan Crops, Fuzhou, China; d Department of Horticulture, College of Tea and Food Science, Wuyi University, Wuyishan, China

**Keywords:** Biocontrol, mitogenome, phylogenetic relationship

## Abstract

*Psyttalia incisi* (Silvestri, 1916), an important solitary opiinae endoparasitoid, plays a crucial role in biological control programs against tephritid pests. In this study, the entire mitochondrial (mt) genome of *P. incisi* was sequenced and characterized. The whole mitogenome of *P. incisi* is 15,188 bp long with a G + C content of 14.80%, and encodes all 37 genes that are typically found in animal mt genomes, which contains 13 protein-coding genes (PCGs), 22 transfer RNA genes (tRNAs), and two ribosomal RNA genes (rRNAs). A maximum-likelihood (ML) tree demonstrates that *P. incisi* is closely related to *Psyttalia lounsburyi*, *Psyttalia humilis*, and *Psyttalia concolor*.

*Psyttalia incisi* (Silvestri, 1916) (Hymenoptera: Braconidae) is a local parasitoid in Fujian Province, China and has become an important biocontrol agent that aim to suppress tephtirid pest populations and therefore reduce economic loss (Yang et al. 2018; Lin et al. 2021). However, to date, few researches concerning its genome information was available. Thus, in this study, we determined the complete mitochondrial genome of *P. incisi* for the first time and analyzed the phylogenetic relationship between *P. incisi* and other braconid wasps.

The adult samples were collected from a pomelo orchard in Zhangzhou City (24.36395°N, 117.3124°E), Fujian Province, China. The voucher specimens were deposited at the Fujian Agriculture and Forestry University with an accession number of 20181001FA (URL: http://zbxy.fafu.edu.cn; contact: Qinge Ji, jiqinge@fafu.edu.cn).

Genomic DNA was extracted using the CTAB extraction method (Vanzyme, Nanjing, China) and a 400-bp insert library was constructed and then sequenced by Illumina Novaseq 6000 platform in 150 bp paired-end read. Raw data was filtered in fastp v.0.20.0 (Chen et al. 2018) resulting in 24,409,678 clean reads, which were then assembled by SPAdes v.3.9.0 software (Bankevich et al. 2012) and annotated by MITOS web server (Bernt et al. 2013).

The length of whole mitochondrial genome of *P. incisi* is 15,188 bp, which contains 13 protein-coding genes (PCGs), two ribosomal RNA genes (rRNAs), 22 transfer RNA genes (tRNAs), and a putative control region (CR). The base composition of the mt genome is 40.33% for A, 8.53% for G, 6.27% for C, 44.87% for T, with a total A + T content of 85.20%, which is heavily biased toward A and T. For the PCGs, six genes (*cox2*, *atp8*, *nad3*, *nad5*, *nad4l*, *nad6*) had an initiation codon of ATT, five (*cox1*, *cox3*, *atp6*, *cob*, *nad4*) had ATG, and two had ATA (*nad1*, *nad2*). All PCGs had the stop codon TAA, except for *atp8* and *nad3* which contained TAG.

We analyzed the nucleotide sequences of 13 PCGs using the Maximum-likelihood (ML) and Bayesian Inference (BI) approaches to understand the phylogenetic relationship of *P. incis*i with 11 other species belonging to the family Braconidae. Phylogenetic analyses were performed with PhyloSuite (Zhang et al. 2020) that was used to conduct, manage and streamline the analyses with the help of several plug-in programs: 13 sequences were aligned in batches with MAFFT (Katoh and Standley 2013) using default parameters, refined using the codon-aware program MACSE v. 2.03 (Ranwez et al. 2018) and removed ambiguously aligned fragments of 13 alignments using Gblocks (Talavera and Castresana 2007). ModelFinder (Kalyaanamoorthy et al. 2017) was used to select the best-fit partition model (Edge-linked) using AICc criterion. Maximum likelihood phylogenies were inferred using IQ-TREE under the model automatically selected by IQ-TREE ('Auto' option in IQ-TREE) for 20,000 ultrafast (Minh et al. 2013) bootstraps, approximate Bayes test (Anisimova et al. 2011), as well as the Shimodaira–Hasegawa–like approximate likelihood-ratio test (Guindon et al. 2010). Bayesian Inference phylogenies were inferred using MrBayes 3.2.6 under partition model (2 parallel runs, 5,000,000 generations), in which the initial 25% of sampled data were discarded as burn-in. The phylogenetic trees indicated that *P. incisi* clustered with *Psyttalia concolor*, *Psyttalia humilis* and *Psyttalia lounsburyi* as a separated clade. The result further confirmed that *P. incisi, P. concolor*, *P. humilis, P. lounsburyi, Diachasmimorpha longicaudata* and *Fopius arisanus* were close to each other, as they all belong to the subfamily Opiinae. So far, there are few studies related to the genome analysis of *P. incisi*, and therefore this study would further clarify the phylogenetic relationship of the Braconidae family and provide valuable information for further studies (Figure 1).

**Figure 1.**
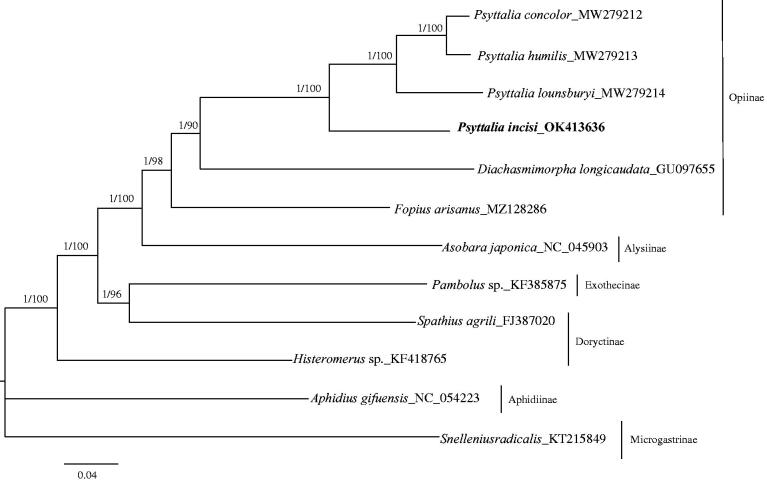
Phylogenetic relationships among subfamilies of the Braconidae inferred from nucleotides of 13 PCGs using Bayesian and maximum-likelihood (ML) methods (GenBank accession numbers provided). The Bayesian posterior probabilities (PP) and bootstrap support (BS) are marked besides the nodes.

## Ethical approval

This study was completed in the laboratory under the premise of the ethical standards, so it was exempted from any ethical approval and didn’t need any permissions to carry it out.

## Data Availability

The complete mitochondrial genome sequence of *P. incisi* is deposited in the GenBank database under the accession number OK413636. The associated BioProject, SRA, and Bio-Sample numbers are PRJNA769539, SRR16767017, and SAMN22120162, respectively. The Web link is https://www.ncbi.nlm.nih.gov/.
